# Short Communication: Tumoricidal activity of lauryl gallate towards chemically induced skin tumours in mice

**DOI:** 10.1038/sj.bjc.6600805

**Published:** 2003-03-18

**Authors:** E Ortega, M C Sadaba, A I Ortiz, C Cespon, A Rocamora, J M Escolano, G Roy, L M Villar, P Gonzalez-Porque

**Affiliations:** 1 Servicios de Inmunologia, Hospital Ramón y Cajal, Ctra. Colmenar Km 9, Madrid 28034, Spain; 2 Cirugia Experimental, Hospital Ramón y Cajal, Ctra. Colmenar Km 9, Madrid 28034, Spain; 3 Anatomia Patológica, Hospital Ramón y Cajal. Ctra. Colmenar Km 9, Madrid 28034, Spain

**Keywords:** lauryl gallate, antioxidants, protein tyrosine kinase inhibitors, antitumoral agents, food additives

## Abstract

Lauryl gallate (antioxidant food additive E-312) prevents the formation of dimethylbenzanthracene-induced skin tumours in mice, and kills, selectively, tumoral cells on established tumours. This results in total remission, after topical application of the compound on the tumoral mass, without affecting the surrounding tissue.

We have reported that triphenols such as gallic acid (3,4,5-trihydroxybenzoic acid) and its esters (methyl, propyl (E-310), octyl (E-311) and lauryl (E-312)) inhibit protein tyrosine kinases (PTKs) (Lázaro *et al*, 1995; Palacios *et al*, 2002). Similarly to other PTK inhibitors such as genistein (Uckun *et al*, 1995), herbymicin (Otani *et al*, 1993), staurosporin (Bossy-Wetzel *et al*, 1998), tyrphostins (Burger *et al*, 1995), etc., they show an antiproliferative effect on tumoral cell lines (Serrano *et al*, 1998). The most hydrophobic of the alkyl gallates (octyl and lauryl) exhibit a proapoptotic potency between 50 and 250 times higher than gallic acid for the lines tested (Serrano *et al*, 1998). The study of lauryl gallate (LG) on the pre-B cell lymphoma Wehi 231 line shows that it inhibits PTKs both in crude extracts and whole cells, disrupts the mitochondrial membrane potential, promotes the efflux of cytochrome *c* to the cytosol, activates the cascade of caspases and induces oligonucleosomal breakdown of DNA (Roy *et al*, 2000). Also these types of compounds seem to show a selectivity for rapidly growing cells, opening the possibility of their study as potential antitumoral agents (Serrano *et al*, 1998). Here, we report that LG not only prevents the formation of chemically induced skin tumours in mice but is also able to kill, selectively, tumoral cells in established tumours.

## MATERIALS AND METHODS

### Chemicals

Lauryl gallate (Fluka), 7,12-dimethylbenzanthracene, phorbol myristate acetate, dimethylsulphoxide, acetone and glycerol were supplied by Sigma-Aldrich Química (Spain).

### Mice

IRC mice (6–8 weeks old) were from the Charles River Laboratories (Saint Aubin les Elbeuf, Rouen, France). All experiments involving ‘*in vivo*’ testing were carried out with ethical committee approval and met the standards required by the guidelines for the welfare of animals in experimental neoplasia (Workman *et al*, 1998).

#### Prevention of tumour formation

Thirty-two IRC mice were individualised and subdivided into four groups (four females and four males per group), as shown in Table 1

**Table 1:** Effect of different doses of LG on the prevention of tumour formation and regression of established tumours

	**Prevention of tumour formation**			**Regression of established tumours**		
**Group**	**LG 2.5**	**LG 10**	**LG 50**	**LG 100**	**LG 250**	**LG 500**
*Sex*						
Male	1/4	2/4	4/4	0/2	2/4	3/3
Female	0/4	2/4	3/4	0/3	2/4	2/4

. The induction of the tumours was performed following the protocol described (Segal *et al*, 1978) with a slight modification. The solvent used was a mixture of dimethylsulphoxide and glycerol (1 : 1), in which all the compounds were soluble. After a single application of 25 *μ*g of 7,12-dimethylbenz[a]anthracene (DMBA) in a volume of 20 *μ*l, and 15 days without treatment, a solution of 5 *μ*g of phorbol-12-myristate-13-acetate (PMA) with different doses of LG (Fluka) was applied topically three times per week. The doses of LG were 0 (controls), 2.5, 10 and 50 *μ*g in a volume of 20 *μ*l per application. After 6–7 weeks of treatment, tumours appeared on 90% of the control mice, and the behaviour of LG was evaluated as shown in Table 1.

#### Regression of established tumours

Twenty-four individualised mice were treated with a single application of 25 *μ*g of DMBA, and after 15 days with no application of compounds, 5 *μ*g of PMA was applied three times a week (6–7 weeks) until the tumour appeared. The mice were subdivided into three different groups (LG 100, LG 250 and LG 500, four males and four females per group), depending on the treatment dose after tumour formation. The volume per application was 20 *μ*l, which was sufficient to cover the whole tumour surface and was applied three times a week for a period of 8 weeks.

## RESULTS AND DISCUSSION

**Figure 1:**
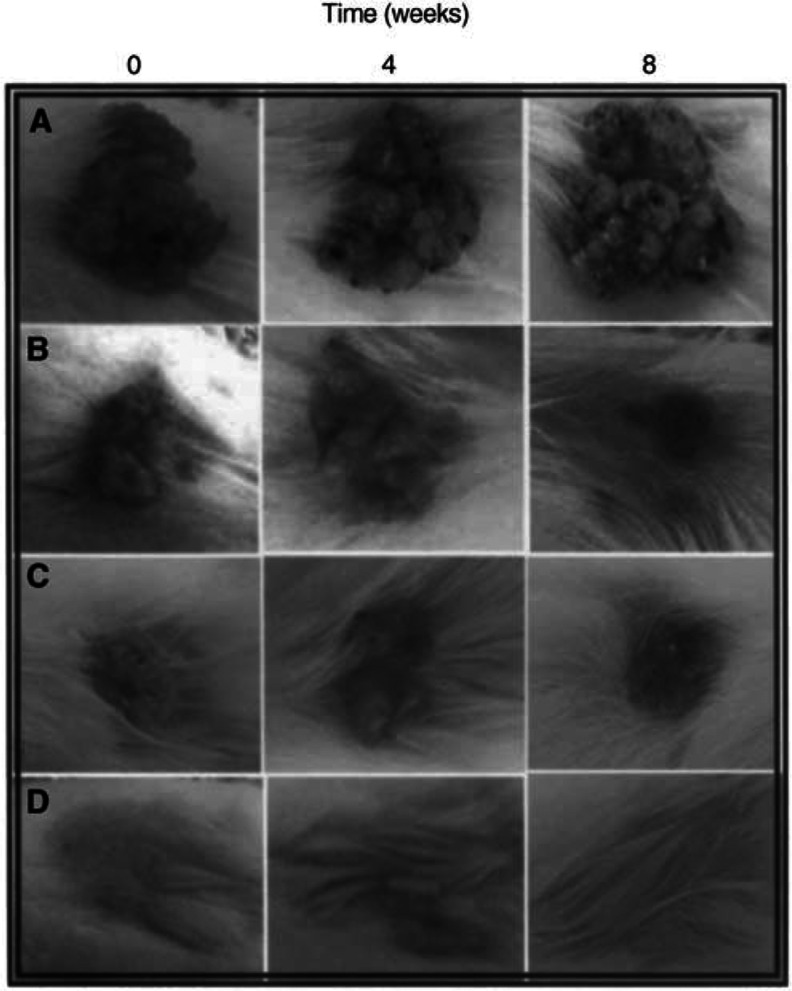
Effect of different doses of LG on the regression of DMBA+PMA induced tumours. (**A**) 100 *μ*g, (**B**) 250 *μ*g, (**C**) 500 *μ*g and (**D**) 500 *μ*g on healthy skin.

To test for the effect of LG on chemically induced skin tumours, we have used the two-stage carcinogenesis model (Segal *et al*, 1978) consisting in the single application of the carcinogen DMBA followed by repetitive applications of the noncarcinogen promoter PMA. We have slightly modified the published procedure by substituting the solvent currently used (acetone) for a mixture of dimethylsulphoxide/glycerol (1 : 1). This results in an easier application and better spreading of the compounds on the mouse skin and avoids the drawback of itching, uneasiness, and sore formation in mice because of the delipidisation produced by the continuous use of acetone. Consequently, mice are easier to handle and are less aggressive. Also, this change does not affect the growth of tumours since more than 90% of mice developed papillomas. Table 1 summarises the results obtained in two types of experiments. In one of them we investigated the effect that increasing amounts of LG added together with the PMA solution have on the prevention of tumour formation. In the other, we studied the effect that topical application of an LG solution has on already established tumours. In both cases, partial inhibition (i.e. tumour size, number of papillomas, etc.) was not considered. Only those mice in which there is complete prevention of tumour formation or total remission of established tumours are recorded in the table. The term ‘full remission’ (arbitrarily defined) refers to those cases in which no papillomas were visually present after 8 weeks of treatment with LG and that if left untreated for a period of up to 3 months they did not exhibit the growth of new papillomas. In both series there is a clear dose–response relation, although the amounts of LG needed for the prevention of tumour formation are roughly 10 times lower than those needed for attaining full regression of established tumours. Figure 1

shows the effect that topical application of the solution of LG has on the regression of tumours. Time 0 is the time at which treatment was installed (all tumours had a developing time, under standard conditions, of around 8 weeks). The treatment was evaluated after 4 and 8 weeks of the initiation (see Materials and Methods). At a dose of 100 *μ*g per application, the effect observed is only partial after 8 weeks (Figure 1A). However, when the dose was increased to 250 *μ*g (Figure 1B) or 500 *μ*g (Figure 1C), full remission was reached, in most cases, after the same period of time. In all cases, it was noticeable that during the treatment, tumours changed in size and shape, passing from a smoother surface at the beginning to a cauliflower-like surface with multiple indentations and pedunculations which later fall without bleeding. However, the surrounding nontumoral tissue is not affected by the treatment. Indeed, the application of the highest dose (500 *μ*g) on healthy mice does not produce any effect during the same period of time (Figure 1D).

**Figure 2:**
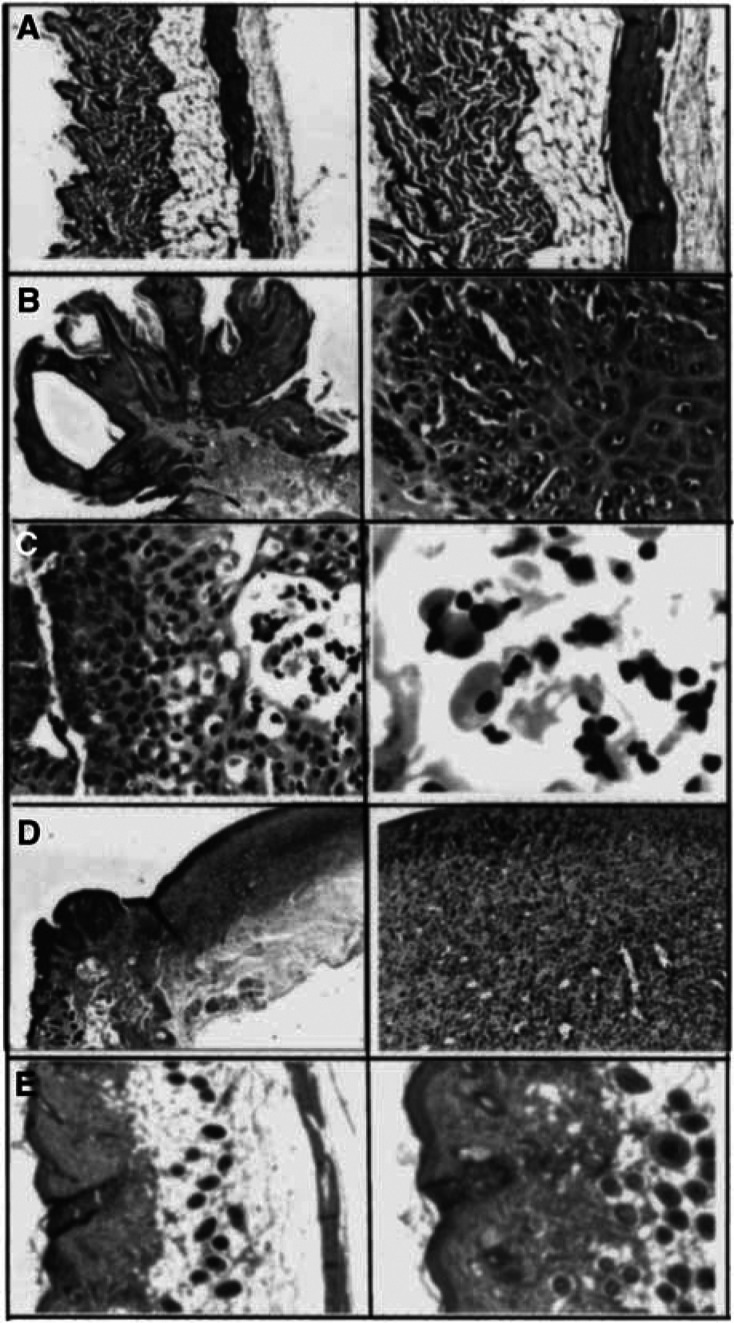
Anatomopathological studies. (**A**) Normal skin, (**B**) untreated tumour, (**C**) treated tumour for 4 weeks with 500 *μ*g of LG, (**D**) skin after tumour regression (treatment with 500 *μ*g of LG for 8 weeks) and (**E**) normal skin treated with 500 *μ*g of LG for 8 weeks.

To analyse the anatomopathological characteristics of the tumours undergoing LG treatment, mice were killed under anaesthesia after different periods of time. Figure 2A

shows the normal skin, without significant lesions, of a healthy mouse. The untreated tumoral mass induced by DMBA and PMA shows an exophytic squamous lesion with a central core. The epidermal surface presents orthokeratotic hyperkeratosis with clumps of red cells and neutrophils. The tumour infiltrates the superficial dermis, and marked nuclear atypia and frequent mitosis are also found (Figure 2B). The characteristics of the tumoral mass after 4 weeks of treatment with LG show features similar to the untreated tumour. However, an increase in the number of apoptotic cells was found (Figure 2C). The residual lesion after 8 weeks of LG treatment (the tumoral mass has already disappeared) shows a deep ulcer penetrating the muscular layer and granulation tissue at the base. Also there is reactive hyperplasia of the epidermal borders of the ulcer (Figure 2D). Finally, the treatment with LG on healthy mice (analogue to Figure 1D) does not produce significant alterations on the skin (Figure 2E).

As a complement or substitution of the traditional armamentarium against tumoral growth and chemoprevention, based mainly in cytotoxic chemotherapy, because of its toxicity, new approaches dealing with the manipulation of signal transduction pathways have been the centre of research during the last years. Protein tyrosine phosphorylation is an early and key step activated by receptor and nonreceptor PTKs which have been implicated in carcinogenesis, tumour progression and metastasis when their activities are not properly regulated (Kolibaba and Druker, 1997; Porter and Vaillancourt, 1998; Blume-Jensen and Hunter, 2001). An intensive search for inhibitors of these enzymes has proven to be fruitful and some of these compounds are already at the preclinical and clinical stages (Levitt and Koty, 1999). One would expect that competitive inhibitors towards the tyrosine (acceptor substrate) could be more efficient than inhibitors towards ATP (donor substrate) because of the high intracellular ATP concentration (in the mM range). However, most of the current strategies are designed to inhibit the ATP binding by specific PTKs which are overexpressed in certain tumours (i.e. imatinib mesylate (formerly STI571) for Bcr-Abl in chronic myeloid leukaemia (Thiesing *et al*, 2000); ZD1839 (Iressa) for epidermal growth factor receptor (Albanell *et al*, 2002) or WHI-PI 131 for Janus 3 kinase in acute lymphobastic leukaemia (Uckun *et al*, 1999), among others). However, sometimes the aberrant PTK is not identified or the growth of the tumour may depend on the concerted action or partial contribution of several PTKs. In such cases, the use of a general nonspecific PTK inhibitor would be worthwhile considering. It may be argued that these kinds of inhibitors might have a significant effect on normal cells as well. Nevertheless, the activity of PTKs in normal cells is usually in the ‘off’ state, which is switched to the ‘on’ state by means of specific ligand, activation of transduction cascades, aberrant overexpression of the oncogenic protein or expression of a mutant protein that escapes the tight regulation of these enzymes. In our opinion, the use of LG (or other alkyl gallates) as one of such inhibitors could be worth investigating considering the following.

*First*, LG inhibits PTKs in whole cells, crude extracts and purified form (PTK c-Src) without inhibiting serine/threonine kinases (Palacios *et al*, 2002) and are not affected by high ATP concentrations. This compound and other gallic acid derivatives are able to induce apoptosis in tumoral cell lines showing selectivity for rapidly growing cells (resting lymphocytes can stand much higher concentrations than mitogen-stimulated lymphocytes (Serrano *et al*, 1998).

*Second*, as shown in this report, LG is able to prevent the tumour formation of skin tumours induced by DMBA and PMA in mice and is able to induce full regression after topical application, on already established tumours.

*Third*, because of their low toxicity, alkyl gallates have been widely used as food additive antioxidants for over 50 years. Thus, much of the work on their metabolism, toxicological effect and pharmacokinetic studies has already been performed. Long-term toxicity studies in rats and mice have shown that no effects were observed at doses as high as 1000 mg kg^−1^ feed. In 1976, the FAO/WHO Joint Expert Committee on Food Additives (JECFA) established an acceptable daily intake (ADI) for man of 0.2 mg kg^−1^ body weight as a sum of propyl, octyl and LG (Van der Heijden *et al*, 1986).

Also, their simple chemical structures and availability in unlimited amounts at a very low commercial cost are important aspects to consider.
